# Awareness of the mythical causes of cancer in central Uganda: a community cross-sectional survey

**DOI:** 10.1007/s10552-026-02249-7

**Published:** 2026-09-11

**Authors:** Nicholas Matovu, Helen G. Coleman, Gerald Mutungi, Michael Donnelly, Brian T. Johnston, Maurice B. Loughrey, Alfred Jatho, Noleb M. Mugisha, Charlene M. McShane

**Affiliations:** 1 Center for Public Health, Queen’s University Belfast, Belfast, UK; 2 Patrick G Johnston Centre for Cancer Research, Queen’s University Belfast, Belfast, UK; 3 Department of Non-Communicable Diseases, Ministry of Health, Kampala, Uganda; 4 Deartment of Gastroenterology, Belfast Health & Social Care Trust, Belfast, UK; 5 Department of Cellular Pathology, Belfast Health and Social Care Trust, Belfast, UK; 6 Uganda Cancer Institute, Kampala, Uganda; 7 King Ceasor University, Kampala, Uganda

**Keywords:** Cancer awareness, Mythical causes, Cancer prevention, Uganda

## Abstract

**Purpose:**

Misconceptions about cancer causes may undermine prevention efforts, yet they remain poorly studied in Africa. This study assessed awareness of mythical cancer causes among adults in central Uganda.

**Methods:**

A community-based cross-sectional survey was conducted among 428 adults (≥ 18 years) in central Uganda using multistage random sampling. Participants completed a modified Cancer Awareness Measure-Mythical Causes Scale. Good awareness was defined as correctly identifying at least 10 of 20 mythical causes. Descriptive and non-parametric analyses examined awareness and associated factors.

**Results:**

Participants had a mean age of 36.5 ± 14.5 years, and 61.2% were female. Awareness of mythical cancer causes was extremely low; only 1.6% correctly identified at least half of the items, with a mean score of 2.4/20 (12%). The most commonly endorsed myth was that “eating food cooked in a polythene bag” causes cancer (95.3%). Lower awareness was associated with older age, being married, and lower education, while having a relative with cancer was associated with better recognition of myths (p < 0.05).

**Conclusions:**

Misconceptions about cancer causes were widespread in this Ugandan population. Cancer awareness initiatives should promote evidence-based risk factors while dispelling common myths, particularly among older and less-educated populations, to strengthen cancer prevention.

## Introduction

Several modifiable risk factors such as unhealthy diets, excessive alcohol consumption, tobacco use and physical inactivity, as well as certain infections, are known to predispose individuals to cancer [1]. Research on the global burden of disease has shown that about 44% of all cancer deaths globally are attributable to potentially modifiable risk factors [2]. Despite this, population-level awareness of known cancer risk factors remains mixed and differs between countries. For instance, high awareness was found for some cancer risk factors (like smoking and ionising radiations) in high-income populations in Sweden and Denmark[3] while, in some reports from low- and middle-income settings, [4, 5] awareness remains relatively poor. Population-based studies in both low- and high-income settings have reported that smoking/tobacco use and excessive alcohol consumption are among the most well-recognised cancer risk factors among the general population [4, 6, 7], while infections are less frequently identified as possible causes [6].

Mythical beliefs about cancer also persist and may undermine prevention and control efforts [8]. Mythical causes of cancer are exposures or agents perceived by the public as actual causes, despite lacking scientific consensus for a causal link [9]. In many African nations, including Uganda, such myths are tied to culture and traditional beliefs [10]. For example, in certain African traditions, witchcraft has been perceived as a cause of ill-health, particularly for certain non-communicable diseases [11]. Believing in ‘incorrect causes of cancer’ (according to current scientific knowledge) can mislead public understanding, foster stigma and mistrust, and prompt individuals to seek help from traditional healers or remedies rather than medical sources. This can delay access to appropriate healthcare, screening and treatment, ultimately undermining cancer prevention and control efforts [12, 13].

The International Agency for Research on Cancer (IARC) through its monograph system [14] periodically reviews evidence concerning various agents (such as biological, chemical, occupations etc.) and their causation of cancer, classifying them as Group 1, 2 A, 2B and 3. According to the current classification, Group 1 agents are those that have sufficient human, animal and mechanistic data on their association with any cancer type in humans. Classification of an agent under any other group rather than Group 1 does not completely mean that that agent is not carcinogenic, but the evidence is not yet sufficient to classify it as such. Therefore, the so called “mythical causes of cancer” in this study does not rule out total causation of cancer by the agents listed as so, but rather this study relies on the fact that the IARC has not classified them as Group 1.

Previous studies globally have illustrated the persistence of mythical beliefs for different cancer types. In a multi-site study led by Moodley et al. (2020) in South Africa and Uganda, some women attributed breast cancer to “*putting money in a bra*” (21.1%) and cervical cancer to “*poor personal hygiene*” (93% on prompted questioning and 33.7% on unprompted questioning). [5] Similarly, in Hong Kong, many Chinese women perceived contaminated foods and environmental pollution as primary risk factors for cancer in general and, more specifically, cervical cancer [15].

While considerable research has focused on awareness of established cancer risk factors, little attention has been given to mythical causes of cancer, yet this may offer a divergent perspective on the public understanding of cancer. This gap is particularly relevant in Africa, where cultural norms and traditional beliefs often distort perceptions of cancer causation. In Uganda, a key knowledge gap exists regarding awareness of mythical cancer causes in general, unlike the site-specific focus of previous work by Moodley and colleagues [5]. This study therefore aimed to assess public awareness of mythical causes of cancer in a central Ugandan population and examine associated demographic and lifestyle factors. Findings from this research are valuable to decision makers in cancer programmes, particularly in designing sensitisation and awareness campaigns aimed at strengthening public knowledge and understanding of cancer.

## Methods

### Study design and site

This community-based cross-sectional survey was conducted in central Uganda between August and October 2022, as part of a larger study assessing colorectal cancer (CRC) awareness [16]. Participants were recruited from Kampala and Mpigi districts to capture diverse sociodemographic and ethnic populations. Kampala, Uganda’s capital, is divided into five administrative divisions and has a population of approximately 1.8 million according to the 2024 national census [17]. Mpigi District is predominantly rural, with an estimated population of 322,131[17], comprising seven sub-counties.

### Study population and sampling

The study included adults aged 18 years and older residing in Kampala and Mpigi districts. Participants were selected using a multistage random sampling approach involving the selection of sub-counties, parishes and households. Details of the sampling procedures have been published elsewhere [16]. Individuals with a current or previous cancer diagnosis, those unable or unwilling to provide informed consent, and those with physical or cognitive impairments that would prevent participation were excluded.

The mythical causes module was embedded within a pre-planned, population-based CRC awareness survey [16], which estimated a sample size of 421 participants. The module was administered to the same survey participants during the same data-collection exercise. The present analysis also used the same sampling frame given that it was designed to estimate awareness levels rather than evaluate an intervention.

### Data collection

Data were collected through face-to-face interviews using a pretested, semi-structured questionnaire that included demographic characteristics, lifestyle behaviours, awareness of mythical cancer causes, among other domains. Local village council leaders assisted with household identification and participant selection. Interviews were conducted by the first author (NM), who is fluent in English and Luganda. Written informed consent was obtained from all participants before interviews. Data were entered into Epi Info™ version 7.2.5.0 (Centers for Disease Control and Prevention, Atlanta, USA).

### Variables and their measurements

#### Sociodemographic and lifestyle variables

Sociodemographic information included age, sex, marital status, education, occupation, household size, duration of residence and distance to the nearest health facility. Lifestyle variables included smoking status, alcohol consumption, physical activity, and personal or family history of cancer. All these were based on self-reported responses.

#### Mythical causes of cancer

Awareness of mythical causes of cancer was assessed using a modified version of the validated *Cancer Awareness Measure MYthical Causes Scale (CAM-MYCS)*[9]. The original 12-item scale was expanded to 20 items by adding eight myths identified from the literature and local beliefs about cancer causes in Uganda. Participants rated each statement using a five-point Likert scale. Following the original scoring approach [18], “strongly disagree” and ”disagree” were scored as correct (1 point), whereas ”agree”, ”strongly agree” and ”not sure” were scored as incorrect (0 points). Total scores ranged from 0 to 20, with higher scores indicating greater awareness that the listed items are not established cancer causes.

### Statistical analysis

Data were analysed using STATA version 16 (StataCorp, College Station, TX, USA). Participant characteristics were summarised using descriptive statistics. Categorical variables were compared using Chi-square or Fisher’s exact tests, while continuous variables were compared using Mann–Whitney U test.

Participants identifying at least 10 of 20 items (≥ 50%) correctly were classified as having good awareness, whereas those who identified below 10 were classified as having poor awareness. The ≥ 50% cut-off for classifying participants as having good awareness was established based on half of the total number of questions (i.e. 10/20), following debate and consensus among the research team. The proportion endorsing each mythical cause (agreeing or strongly agreeing that it causes cancer) was calculated and presented graphically.

Because CAM-MYCS scores were non-normally distributed and violated the assumption of homogeneity of variances, associations between awareness scores and participant characteristics were assessed using the Mann–Whitney U and Kruskal–Wallis tests. Significant Kruskal–Wallis results were followed by Conover-Iman post hoc pairwise comparisons with Bonferroni correction for multiple testing. Statistical significance was set at p < 0.05.

## Results

Of the 460 participants initially contacted to take part in the study, 428 agreed to participate (response rate = 93%). The majority of participants were female (61.2%, n = 262). Participants’ age ranged from 18 to 82 years with an overall mean (SD) age of 36.5 (± 14.5) years, with no statistically significant difference among males and females observed (36.2 vs. 36.6 years, p = 0.78). The participants’ sociodemographic and lifestyle characteristics are summarised in Table 1.

**Table 1:** Association between awareness of mythical causes of cancer and different sociodemographic and lifestyle variables among a sample of the central Ugandan population

Parameter	Overall	MYCS scores Mean ± SD	MYCS scores Median (IQR)	p^†^	P–value (Pairwise comparison) ^**§**^
Overall	428 (100)	2.4 ± 2.69	2 (3)		
Age, years (mean ± SD)	36.5 ± 14.5			< 0.001	
<30	23.5 ± 3.2	3.0 ± 3.0	2 (3)		(Ref)
30–59	41.3 ± 8.3	2.1 ± 2.5	1 (3)		< 0.001
60 +	68.0 ± 7.0	1.4 ± 1.4	1 (3)		0.002
Sex				0.21	
Male	166 (38.8)	2.5 ± 2.6	2 (3)		
Female	262 (61.2)	2.3 ± 2.7	1.5 (3)		
District				0.09	
Mpigi	260 (60.7)	2.7 ± 2.9	2 (3)		
Kampala	168 (39.3)	2.0 ± 2.1	1 (3)		
Marital status				<0.001	
Single	129 (29.2)	3.2 ± 3.4	2 (3)		(Ref)
Married	125 (29.2)	2.0 ± 2.1	1 (3)		0.006
Cohabiting	119 (27.8)	2.6 ± 2.4	2 (3)		0.89
Others	59 (13.8)	1.2 ± 1.9	0 (2)		< 0.001
Occupation				0.35	
Salaried	32 (7.5)	3.3 ± 3.9	2.5 (4)		
Self/casual	274 (64.0)	2.3 ± 2.7	2 (3)		
Unemployed	87 (20.3)	2.2 ± 2.4	2 (3)		
Students	30 (7.0)	2. 7 ± 2.2	2 (3)		
Retired	5 (1.2)	2.2 ± 1.3	2 (2)		
Education				< 0.001	
No school	19 (4.5)	1.4 ± 1.6	1 (2)		(Ref)
Primary	185 (43.2)	1.8 ± 2.4	1 (3)		1.000
Secondary	179 (41.8)	3.1 ± 2.9	2 (4)		0.009
Tertiary (non-degree)	36 (8.4)	2.7 ± 2.7	2 (3)		0.22
Tertiary (degree)	9 (2.1)	1.9 ± 2.2	1 (4)		1.00
Household members				0.93	
1–3	161 (37.6)	2.5 ± 2.9	2 (4)		
4–6	178 (41.6	2.3 ± 2.5	2 (2)		
7+	89 (20.8)	2.4 ± 2.7	2 (3)		
Distance from nearest HF				0.57	
Less than 1 km	120 (28.0)	2.4 ± 2.8	2 (3)		
About 1 km	126 (29.5)	2.4 ± 2.6	2 (2)		
About 2–4 km	123 (28.7)	2.3 ± 2.7	2 (3)		
5 or more km	59 (13.8)	2.7 ± 2.6	2 (4)		
Current smoking status				0.64	
Daily/occasionally	23 (5.4)	2.1 ± 2.3	2 (3)		
Not at all	405 (94.6)	2.4 ± 2.7	2 (2)		
Current alcohol drinking				0.53	
Yes	171 (39.9)	2.3 ± 2.6	2 (3)		
No	257 (60.1)	2.5 ± 2.7	2 (3)		
Physical activity status				0.46	
No activity/sedentary	144 (33.64)	2.2 ± 2.4	2 (3)		
Light	19 (4.44)	3.8 ± 4.5	2 (4)		
Moderate	66 (15.42)	2.3 ± 2.5	1 (3)		
Vigorous	199 (46.50)	2.5 ± 2.7	2 (4)		
Relative/FM with any cancer				0.002	
Yes	88 (20.6)	3.0 ± 2.7	2 (4)		
No	340 (79.4)	2.2 ± 2.7	1 (3)		

*HF* Health facility, *FM* family member; ^**†**^ P-value for Kruskal Wallis test or Mann Whitney U test for comparison across three and two groups, respectively. ^§^Pairwise comparison p-value obtained by the Conover-Iman tests, with Bonferroni adjustment

### Scale reliability of the CAM-MYCS

The 20 questions that were used to measure awareness of the mythical causes of cancer had a good internal consistency reliability (Cronbach’s alpha = 0.87). The internal consistency of the 12 original questions as used by Smith et al*.*[9], was lower than that of the 20 questions, but still satisfactory (Cronbach’s alpha = 0.82).

### Endorsement of the different mythical causes of cancer

**Fig. 1:**
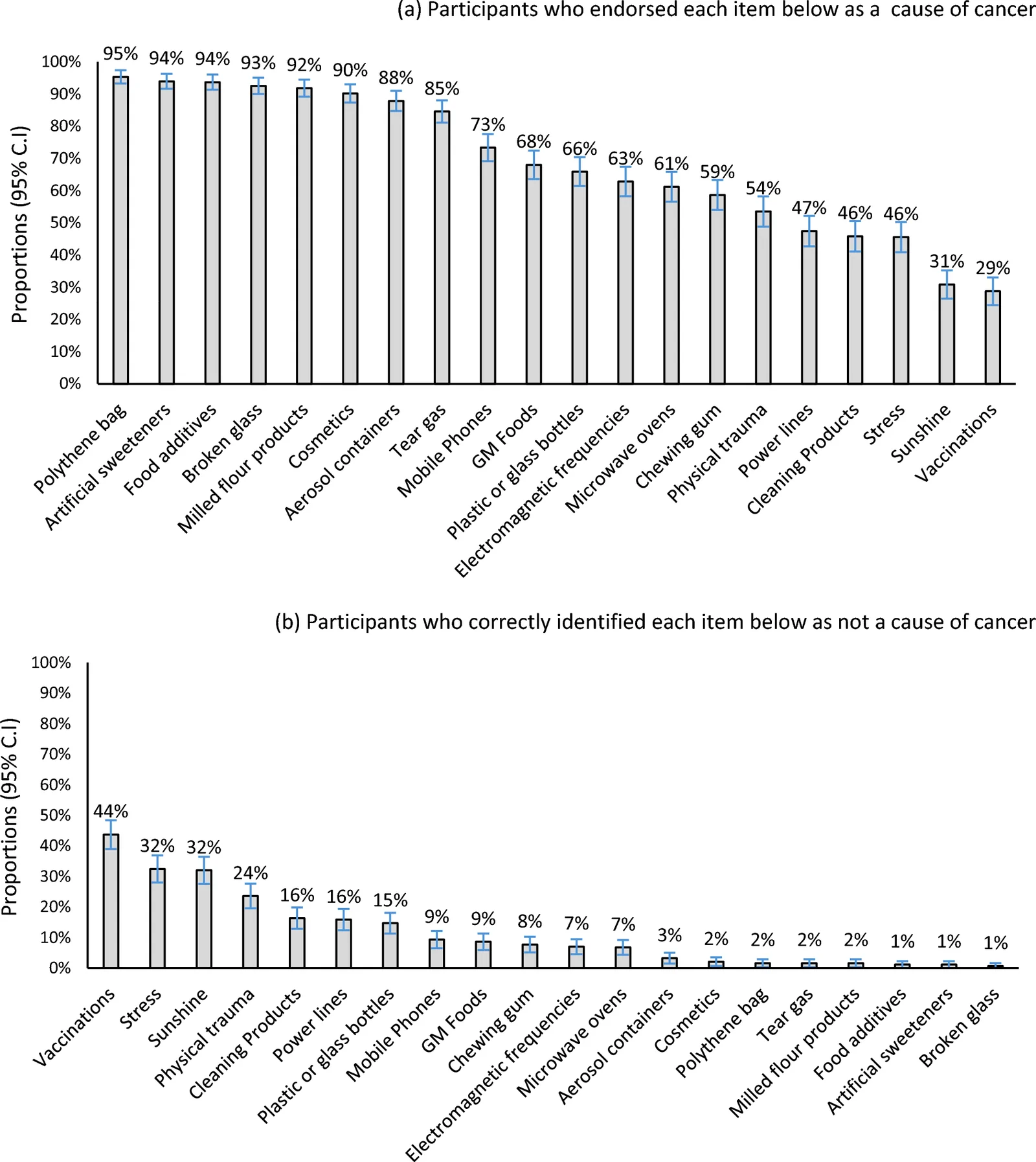
Proportion of participants **a** endorsing the items listed as causes of cancer and **b** correctly identifying the items listed as not a cause of cancer

Figure 1a demonstrates the proportion of participants who endorsed each of the items listed as a cause of cancer. Endorsement was generally higher across all items asked, meaning that participants were more likely to agree that the items listed were a true cause of cancer. The most endorsed items included “eating food cooked/steamed using a polythene bag” and “eating food containing artificial sweeteners”, which were endorsed by 95.3% (95%C.I: 92.8%, 96.9%) and 93.9% (95%C.I: 91.2%, 95.8%) of the participants respectively. The least endorsed items included “receiving vaccinations” (28.7%; 95%C.I: 24.6%, 33.2%) and “staying in the sunshine for long hours” (30.8%; 95%C.I: 26.6%, 35.4%).

### Recognition of the different mythical causes of cancer

Figure 1b shows the proportion of participants who correctly identified the items listed as not being causes of cancer (i.e. mythical cancer causes). The most frequently identified mythical cause items were “receiving vaccinations” (44%, 95%C.I: 39.0%, 48.4%), “stress” (32.4%; 95%C.I: 28.2%, 37.1%) and “staying in sunshine for long hours” (32.0%; 95%C.I: 27.7%, 36.6%). Conversely, the least identified mythical cause items were “drinking or eating food containing pieces of broken glass” (0.70%; 95%C.I: 0.23%, 2.16%) and “eating food containing artificial sweeteners” (1.17%; 95%C.I: 0.49%, 2.78%).

### Overall awareness of the mythical causes of cancer

The overall awareness of the mythical causes of cancer was very low where only seven out of the 428 participants (1.64%, 95%C.I: 0.78%, 3.39%) could correctly identify at least 10 of the 20 mythical causes asked as not actual causes of cancer. On average, participants were able to correctly identify approximately two of the 20 mythical causes asked (mean MYCS score ± SD = 2.4 ± 2.6).

### Association between awareness of the mythical cancer causes and the different sociodemographic and lifestyle variables

Of the variables investigated, age, marital status, education level and cancer family history status were significantly associated with awareness of the mythical causes of cancer (Table 1). Pairwise comparisons showed that older participants (30–59 years and aged 60+ years) demonstrated significantly lower mean MYCS scores compared to their young counterparts (aged < 30 years). Married individuals (mean MYCS score: 2.0) and those who were in the others category (mean MYCS score: 1.2) had significantly lower MYCS scores compared to their single counterparts (mean MYCS score: 3.2). Compared to participants who did not attend school (mean MYCS score: 1.4), participants who had attained a secondary level qualification (mean MYCS score: 3.1) had significantly higher MYCS scores (p = 0.009). Lastly, participants with a family member or relative with cancer (mean MYCS score: 3.0) demonstrated significantly higher MYCS scores compared to their respective counterparts with no cancer family history (mean MYCS score: 2.2), p = 0.002.

## Discussion

This study assessed awareness to the mythical causes of cancer in a central Ugandan population. Results generally demonstrated low awareness levels to the mythical causes of cancer, with participants only able to correctly identify on average two out of 20 mythical items as not established cancer causes. On the other hand, consuming artificial sweeteners and eating food cooked in a polyethene bag were the most common myths endorsed as cancer causes.

There is limited research on the awareness of the mythical cancer causes in LMICs, as well as globally. In the few studies available, awareness of the mythical causes of cancer has been found to be low. In a population-based study in Pakistan, participants were able to correctly identify 46% of 13 proposed myths as not being cancer causing (i.e. about 6/13) [19], which is in contrast to 12% (2.4/20) of participants in the present study. When compared to high-income countries, participants in this study still reported a lower recognition of mythical cancer causes. A cross-sectional study in England [18] reported that 36% of 12 mythical causes were correctly identified as such (i.e. about 4/12). In African communities, where cultural beliefs are deeply rooted like in Uganda, there is a unanimous belief that modern technology from Western countries is a danger to African culture and is, in most cases, regarded as harmful, including to health [20]. As most of the mythical cancer causes assessed in this study had a link with modern Western technology such as “artificial sweeteners”, “polythene bags”, participants may have regarded them as harmful and potentially carcinogenic. To support this interpretation, items such as “stress” and “physical trauma” were some of the least endorsed mythical causes of cancer, perhaps as they are not linked with any Western technology. However, we can also not rule out the fact that there is a certain degree of misinformation on health issues across the media that likely triggers a lot of myths in the public [21], and this could be one of the reasons behind the generally low awareness on mythical cancer causes across several other countries including and beyond Uganda. In the same way, our findings generally align with the notion that the public often believes “everything causes cancer,” as reported by Paytubi [8], who found nearly half of respondents endorsing this view, highlighting the need for intensified cancer awareness efforts.

This study also found young participants in a better position to correctly recognise the mythical causes of cancer compared to their older counterparts. This finding is in line with previous research in the UK, where age was negatively associated with awareness scores on mythical cancer causes [18]. Whereas MYCS scores were generally low in this population, being single, as opposed to married, was associated with higher awareness on the mythical causes of cancer in our population. Shahab and colleagues, in their UK cross-sectional study, did not find a significant association between marital status and awareness on the mythical causes of cancer [18]. It can be assumed that married people are more likely to share information amongst themselves, and with fellow family members/relatives, which exposes them to a certain risk of misconceptions. For instance, one study that assessed cancer risk information sharing in the Jewish population found that closeness among family members facilitated information sharing and encouragement of genetic testing for familial cancer mutations [22]. Whereas married people are also likely to be knowledgeable on different issues concerning their health because of such information sharing, a certain risk of health misinformation cannot be ruled out. We also found that having a family member or relative with cancer was significantly associated with better awareness of the mythical causes of cancer. However, Munir et al*.,* in their cross-sectional study in Pakistan, did not find an association between MYCS scores and having a relative or family member with cancer [19]. Further rigorous studies are necessary to assess this association.

### Study strengths and limitations

Our novel study presents findings on the awareness of non-site-specific mythical causes of cancer in Uganda, contributing to the literature on general cancer awareness in the country. The internal consistency reliability of our 20-item modified CAM-MYCS tool in a low-income Ugandan context was found to be satisfactory and may be adapted for use in similar settings.

This study has limitations. Its cross-sectional nature means established relationships cannot be interpreted as causal. Most responses were based on participants’ recall, which may have introduced recall bias. The generalisability of these findings to the entire Ugandan population cannot be guaranteed due to cultural differences across regions. Again, some of the mythical items listed in this study may appear debatable as to their causation of cancer. However, at the time of data collection, the mythical causes of cancer assessed in this study had not been documented to lead to carcinogenesis by the IARC. For instance, our study included “eating food cooked in a polythene bag” as one of the mythical causes of cancer. In Uganda, polythene bags made from polyethylene are commonly used for carrying foods and groceries and on rare occasions, some people use them for steaming local dishes such as “matooke”. It may appear ambiguous for this to be listed as a mythical cause, given that some plastics, particularly when heated to high temperatures, can leach chemicals into food that are harmful to health [23]. However, polyethylene itself has not been classified by IARC as a Group 1 carcinogen [24] and bi-products of heat exposure to polyethylene bags during cooking, such as phthalates [25], are also not classified as such [26]. Similarly, the inclusion of artificial sweeteners in our study may also appear debatable. However, according to the IARC, one of the most widely used artificial sweeteners, aspartame, is classified as Group 2B, meaning it is possibly carcinogenic to humans based on limited evidence in humans and supporting mechanistic data [27]. If future research provides stronger evidence that changes these classifications, our modified tool will need to be updated to reflect the new scientific consensus. The inclusion of “prolonged sun exposure” as a mythical item in our study is purely contextual given the fact that Black African populations experience relatively lower incidence of sun-related skin cancers compared to lighter-skinned populations [28], rather than a complete lack of scientific awareness [29]. Nonetheless, our findings provide insight into community awareness of mythical cancer causes, serving as background information for future studies. Finally, participants’ responses may also have been influenced by their engagement with the broader CRC awareness questions, potentially affecting the reporting of perceived mythical causes of cancer.

## Conclusion

This study demonstrated generally low awareness of mythical causes of cancer, with disparities across certain sociodemographic groups in a Ugandan setting. Participants were likely to endorse than recognise most mythical causes of cancer. Cancer awareness efforts should therefore not only focus on sensitising the public about actual cancer causes but also dispel public myths on what are thought to be possible carcinogens, so as to avoid unnecessary anxiety in the population.

## Data Availability

All data relevant for this study is available within the manuscript or as supplementary material uploaded as part of this paper.
